# A landscape-based cluster analysis using recursive search instead of a threshold parameter

**DOI:** 10.1016/j.mex.2016.06.002

**Published:** 2016-07-07

**Authors:** Thomas E. Gladwin, Matthijs Vink, Roger B. Mars

**Affiliations:** a Military Mental Health Research Centre, Ministry of Defense, P.O. Box 90.000, 3509AA Utrecht, The Netherlands; b Brain Center Rudolf Magnus, Department of Psychiatry, University Medical Center Utrecht, Utrecht, The Netherlands; c Departments of Experimental and Developmental Psychology, Faculty of Social Sciences, Utrecht University, Utrecht, The Netherlands; d Donders Institute for Brain, Cognition and Behaviour, Radboud University Nijmegen, Nijmegen, The Netherlands; e Centre for Functional MRI of the Brain (FMRIB), Nuffield Department of Clinical Neurosciences, John Radcliffe Hospital, University of Oxford, United Kingdom

**Keywords:** Recursive clustering, fMRI, Cluster analysis, Threshold-free, Permutation, Recursive, Derivative

## Abstract

Cluster-based analysis methods in neuroimaging provide control of whole-brain false positive rates without the need to conservatively correct for the number of voxels and the associated false negative results. The current method defines clusters based purely on shapes in the landscape of activation, instead of requiring the choice of a statistical threshold that may strongly affect results. Statistical significance is determined using permutation testing, combining both size and height of activation. A method is proposed for dealing with relatively small local peaks. Simulations confirm the method controls the false positive rate and correctly identifies regions of activation. The method is also illustrated using real data.

• A landscape-based method to define clusters in neuroimaging data avoids the need to pre-specify a threshold to define clusters.
• The implementation of the method works as expected, based on simulated and real data.
• The recursive method used for defining clusters, the method used for combining clusters, and the definition of the “value” of a cluster may be of interest for future variations.

## Graphical abstract

**Figure:**
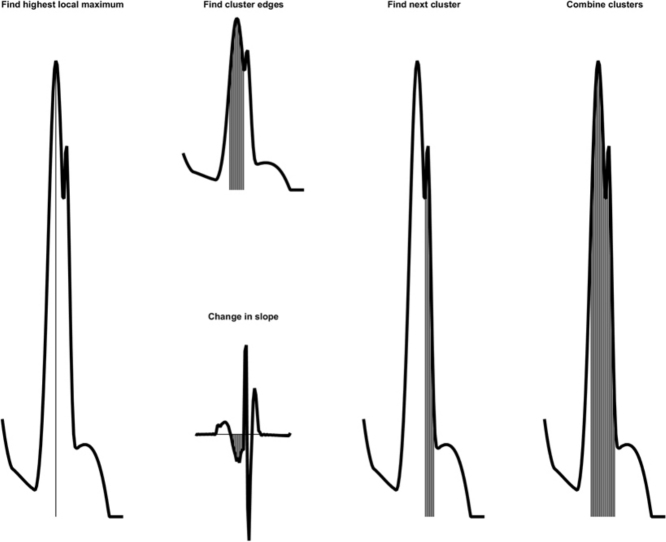

## Method details

The method involves three steps: (1) defining clusters using a recursive search function aimed at detecting an upwards change in the differential of the activation, moving away from a local maximum; (2) defining a condition when to combine adjacent clusters; and (3) permutation tests for the whole-brain maximum of a score per cluster that combines size and activation level. Functions from the SPM toolbox (www.fil.ion.ucl.ac.uk/spm) in Matlab [8] were used for reading and saving files and smoothing data.

Clusters are defined as follows, for a given statistical activation map of −log(*p*) values derived from a T-map or F-map. First, the voxel with the highest value is selected. Then, a recursive function is used to iteratively visit neighboring voxels, then their neighbors, and so on. Voxels are only visited if they are further away from the peak voxel, to avoid doubling back. New voxels are added until the slope in the value from the previous voxel to the new one is more positive than the previous slope. This procedure thus selects the edges of clusters, which start at the peak and at some point must increase their derivative as they drop in the activation landscape. After a cluster is defined, the voxels in that cluster are excluded from further processing and the cluster surrounding the next highest peak in the image is calculated, until no local maxima remain. Local maxima were defined as any voxel for which all eight neighboring voxels had a lower value.

Since local maxima within clusters may occur, depending on the smoothness of the data, the following criterion was used to combine adjacent clusters into a single cluster. If no activation threshold is used at all (which is unnecessary with the current method, although for purposes of speed a liberal threshold of *p* = 0.05 could be used), but some form of cluster-combination is used, this step is particularly important. With a too-liberal combination criterion, the whole “floor” of the activation landscape will be combined into a single very extensive cluster, which may acquire large values during permutation testing under the null hypothesis. In our method, for each cluster the proportion of the edge voxels that border on a different cluster (ProportionConnected) is determined. If this proportion is above zero (that is, if there is any adjacent cluster), it is determined whether to combine the clusters. Two additional values are used for this: The difference between the peak values of the two clusters (PeaksDifference), and the difference between the peak value of the cluster with the lower peak and the mean activation level at the edge-voxels adjacent to the neighboring cluster (SmallerPeakToConnectingEdge). The clusters are combined under the following condition:

PeaksDifference/(PeaksDifference + SmallerPeakToConnectingEdge) ≥ 1 − ProportionConnected

That is: As the amount of connection increases, the more likely the clusters will be combined. In the extreme case, a fully surrounded cluster will always be incorporated into the surrounding cluster. Further, combination is more likely as the lower cluster is less well separated: If the lower cluster’s peak is not much higher than the connecting flank with the higher cluster, it will be combined. The criterion thus differentiates the case of two clearly separated peaks, versus a bump lying on the flank of a larger hill in the activation landscape.

Finally, the significance of clusters is determined using permutation testing [1] to acquire a distribution of whole-brain maximum cluster scores under the null hypothesis. Each cluster is assigned a cluster activation score reflecting its size and level of activation: The sum of values in the cluster. Thus, larger clusters with higher values have higher cluster activation scores. The precise type of permutation may differ depending on the statistics of interest, and requires only the generation of a map of p-values in which spatial dependence has been preserved. For the case of t-tests involving contrast scores, for example, for a number of permutations, each subject’s data can be independently, randomly (with a chance of 0.5) multiplied by −1 or not. Essentially, this reversal is applied to all voxels, thus preserving the spatial dependence between voxels but statistically removing any effect in the data. This random permutation enforces the data being distributed according to the null hypothesis: There can be no systematic, non-random direction of the contrast scores at the group level. For each permutated data set, the maximum activation score over all clusters is stored for the permutation. This results in a null-hypothesis distribution of activation scores of the greatest activation score over the whole brain, rather than a distribution of activation scores over all clusters (which would result in a more liberal test). The activation score taken for the significance criterion is the score above which fewer than 5% of maximum activation scores are found in the null distribution. Clusters in the original, non-permuted statistical map with activation score above this criterion are considered significant at a 5% false positive level, as such scores would be expected less than 5% of observations under the null hypothesis. This results in a mask within which the activation can be considered statistically significant in terms of the cluster-analyses.

The implementation of the algorithm is available as part of the hiro3 fMRI visualization and manipulation tool, available at https://www.tegladwin.com/files/matlab/hiro3.php.

## Additional information and Supplementary material

Here, we present the background of the method and some results of its use. One of the central problems in the whole-brain statistical analysis of brain activation data is the massive multiple testing problem due to the large number of voxels, each of which is associated with a statistical test. Correcting for the number of voxels controls the false positive rate at the cost of drastically reduced power. More permissive methods have been proposed, such as taking a “common sense” combination of a relatively low statistical threshold, in accordance with realistic effect sizes and sample sizes, together with a minimal acceptable cluster size to reduce spurious findings [5]. Such an approach may provide a reasonable compromise, in particular from a perspective on science in which no single study provides extremely strong evidence by itself. However, the clear downside is the inflation of false positive rates to an unknown degree.

A promising alternative route is provided by cluster analyses [1]; e.g., [2], [3], [4], [7], [12]. In these methods, clusters of voxels are tested, rather than separate voxels. This has the intuitive advantage of not punishing imaging techniques for having high resolution, and potentially providing adequate power without any compromise of false positive rates. However, one problem in typical cluster analysis (but see Ref. [7]) is that the researcher must select a threshold of voxel intensity, above which clusters are defined. This may introduce a form of method-snooping [6], [11], and hence increased false positives. On the other hand, an a priori strictly followed threshold could lead to the failure to report some likely true results that just fail to reach that arbitrary threshold.

We therefore developed the current form of cluster analysis, in which there is no threshold used to define clusters. In contrast, clusters are defined purely by the landscape of activation. Further, once clusters are defined the significance can be tested using permutation testing of the summed activation over all voxels in the cluster. This method would thus have the usual attractive features of cluster analyses – controlled whole-brain testing with high power relative to voxel-wise testing – while avoiding the dependence on an arbitrary threshold. The current report shows the performance of the method on simulated data.

The method was tested using simulated and real data. For the simulated data, fMRI contrast maps for 32 simulated subjects were created. The AAL atlas [9] was used as a template to create activation in the voxels labelled as left amygdala with an effect size (contrast score/standard deviation of noise) of 0.8. Normally distributed voxel-wise random noise was generated and smoothed (4 × 4 × 4 mm FWHM), and added to each map. 100 simulations were performed, and 100 permutations were used per simulation. It was tested whether the method would report (only) the clusters containing a simulated effect as significant.

The simulations were also performed for a fixed threshold and cluster-size criterion, of p < 0.005 and N ≥ 20 [5]. A full comparison between the various forms of cluster-dependent methods was beyond the scope of the current paper. The Lieberman and Cunningham method, however, appeared to provide the most relevant comparison. Its practicality likely leads to its relatively frequent use, so that its comparison to a relatively powerful whole-brain correct method may be of particular interest. Further, the current method was most closely related to this kind of cluster analysis, that is: Clusters are defined and statistics performed per cluster, as opposed to, for instance, statistical values being assigned to voxels using cluster-related information. We note that it is an explicit point of Lieberman and Cunningham that the attention given to false positives and false negatives need to be balanced, and agree with this. While it is expected that fewer false positives will occur using the current method, as it by design applies whole-brain correction, we agree that researchers still need to balance and deal with the problems of both types of statistical error. Further, in comparison with voxel-wise whole-brain correction, the comparison is clear a priori: Power will be then far lower, and acquiring results will depend on having very strong effects per voxel.

As a test on real data, a previously published data set was used [10]. The data were from a contrast between a stable-state working memory and a control condition matched for visual input and motor responses, which showed a clear pattern of activation at a p = 0.005, 20 voxel extent threshold [5], in particular at precentral, parietal and basal regions. The results were compared with results using FWE-corrected Threshold-Free Cluster Enhancement, TFCE [7] (via the toolbox created by Prof. C. Gaser; TFCE is closely related to random field theory, Appendix C.2 of Smith and Nichols [7]).

In the simulated data, the method successfully reported significant activation in the amygdala in all of the 100 simulations. 95% of clusters reported as significant were in the simulated region. 80% of the voxels within significant clusters overlapped with the simulated region. Using the Lieberman and Cunningham method with fixed threshold and 20-voxel cluster size, 52% of significant clusters overlapped with the simulated region and 67% of individual voxels were in the simulated region.

**Fig. 1:**
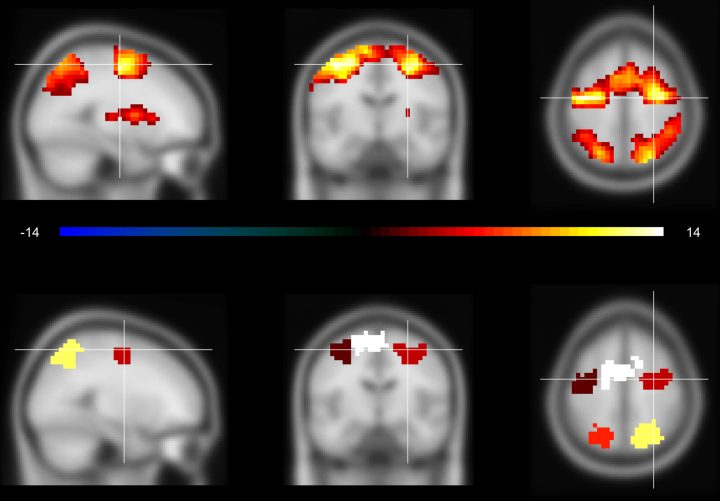
A. Illustration of the map of *t*-values for the working memory—control contrast of a stable-state working memory task [10]. B. Illustration of clusters defined by the recursive algorithm and reported as having significant cluster activation scores by whole-brain corrected permutation testing.

The method also performed as would be expected on the real working memory data [10]. The clearest clusters of activation previously reported were cluster-wise significant, but not the weaker activation in the basal ganglia (Fig. 1). The results were very similar to those acquired via TFCE, suggesting both methods, although quite different in their algorithms, are sensitive to the same kind of clustered effects.

In conclusion, whole-brain analyses are particularly important for exploratory studies, in which it would be undesirable to restrict one’s view to regions of interests. Whole-brain analyses also prevent the problem of methods-snooping in terms of post-hoc selection of “a prior” regions of interest for which a rationale can almost always be thought of. When applied to real data, the method returned those clusters of activation that were visually apparent and had previously been reported based on a threshold aimed at compromise between type I and type II errors [5]. The current method appears to provide a similar reasonable approach to analysis of high-resolution neuroimaging data, with the advantage of certain control of false positives and insensitivity of the details of the method to changes in voxel size.

The appropriateness of the current method depends on the shape and size of clusters of activation that are expected given the theoretical causes of functional activation. “Spotty” results, where scattered voxels show an effect not consistently shared by other voxels in the anatomical region, would not be considered convincing evidence for activation. Cluster analysis using permutation testing formalizes the use of such spatial information. The power of the method will depend on whether the relevant spatial features of activation are adequately exploited in the recursive cluster definition.
